# Optimal Adherence to a Mediterranean Diet and High Muscular Fitness Are Associated with a Healthier Cardiometabolic Profile in Collegiate Students

**DOI:** 10.3390/nu10040511

**Published:** 2018-04-20

**Authors:** Robinson Ramírez-Vélez, Jorge Enrique Correa-Bautista, Mónica Liliana Ojeda-Pardo, Carolina Sandoval-Cuellar, Antonio García-Hermoso, Hugo Alejandro Carrillo, Katherine González-Ruíz, Daniel Humberto Prieto-Benavides, Alejandra Tordecilla-Sanders, Arvydas Martinkėnas, César Agostinis-Sobrinho

**Affiliations:** 1 Centro de Estudios Para la Medición de la Actividad Física CEMA, Escuela de Medicina y Ciencias de la Salud, Universidad del Rosario, Bogotá 111221, Colombia; jorge.correa@urosario.edu.co (J.E.C.-B.); danielprietob@gmail.com (D.H.P.-B.); alesanders_0615@hotmail.com (A.T.-S.); 2 Facultad de Ciencias de la Salud, Universidad de Boyacá, Boyacá 150003, Colombia; mlojeda@uniboyaca.edu.co (M.L.O.-P.); carolinasandoval@uniboyaca.edu.co (C.S.-C.); 3 Laboratorio de Ciencias de la Actividad Física, el Deporte y la Salud, Facultad de Ciencias Médicas, Universidad de Santiago de Chile, USACH, Santiago 7500618, Chile; antonio.garcia.h@usach.cl; 4 Grupo GRINDER, Programa de Educación Física y Deportes, Universidad del Valle, Santiago de Cali 76001, Colombia; hugo.carrillo@correounivalle.edu.co; 5 Grupo Interdisciplinario de Estudios en Salud y Sociedad (GIESS), Institución Universitaria Escuela Nacional del Deporte, Santiago de Cali 76001, Colombia; 6 Grupo de Ejercicio Físico y Deportes, Vicerrectoría de Investigaciones, Universidad Manuela Beltrán, Bogotá DC 110231, Colombia; katherine.gonzalez@docentes.umb.edu.co; 7 Faculty of Health Sciences, Klaipeda University, Klaipeda LT-91274, Lithuania; arvydas.martinkenas@lsmuni.lt (A.M.); cesaragostinis@hotmail.com (C.A.-S.); 8 Physical Education, Physiotherapy and Dance, Federal University of the South of Brazil, Porto Alegre 91501-970, Brazil

**Keywords:** metabolic syndrome, healthy dietary patterns, muscular strength

## Abstract

The aim of the study was to investigate the combined association of adherence to a Mediterranean diet (MedDiet) and muscular fitness (MF) with cardiometabolic health in collegiate students. The present cross-sectional analysis consisted of 1248 (714 females) healthy collegiate students (20.1 ± 2.7 years old). Adherence to a MedDiet was assessed by a KIDMED (Mediterranean Diet Quality Index) questionnaire. Standing broad jump, standing vertical jump, and isometric handgrip dynamometry were used as indicators of MF. The cardiometabolic profile was assessed using the following components: triglycerides, blood pressure, triglycerides, high-density lipoprotein (HDL)-cholesterol, glucose, and waist circumference. Analysis of covariance shows a significant difference in the cardiometabolic profile of both genders between the high MF/low MedDiet and high MF/optimal MedDiet groups, and the low MF/low MedDiet and low MF/optimal MedDiet groups (*p* < 0.001). No difference was found on cardiometabolic profile between high MF/optimal MedDiet and high MF/low MedDiet, both in males and females. Additionally, logistic regression shows that both female (odds ratio (OR) = 2.01; 95% confidence interval (CI): (1.8–3.7); *p* = 0.02) and male (OR = 3.38; 95% CI: (1.9–5.8); *p* < 0.001) participants in the optimal MedDiet/high MF group had the highest odds of expressing a healthier cardiometabolic profile as compared to those in the low MF/low MedDiet group. In conclusion, a combination of high MF levels and optimal adherence to a MedDiet is associated with a healthier cardiometabolic profile; however, high MF levels seem to circumvent the deleterious effects of having a low adherence to a MedDiet.

## 1. Introduction

Despite the existence of different definitions of metabolic syndrome (MetS) [1], most authors agree that MetS is a cluster of three or more risk factors, such as adiposity, high levels of triglycerides, hyperglycemia, low HDL-cholesterol levels, and hypertension [1,2]. The clustering of these risk factors may culminate in adverse outcomes including cancer, Type 2 diabetes, and cardiovascular disease (CVD) [2].

Unhealthy lifestyles are linked to poor cardiometabolic health [2,3,4], accelerating the progression of cardiovascular disorders and increasing mortality [3]. The major risk factors for developing MetS are poor diet and physical inactivity [2]. There is an unequivocal association between physical inactivity and cardiovascular consequences in adults [5]; however, recently there has also been an increased interest in the effects of muscular strength on the cardiometabolic profile [6,7]. Further, certain studies have shown that low muscle strength is strongly associated with a poor health status and a high mortality rate [8,9]. Similarly, in a systematic review and meta-analysis of data from approximately 2 million men and women, we have previously reported that higher levels of muscular strength are associated with a lower risk of mortality in the adult population [7].

Poor dietary habits are also important with regard to the onset and advancement of metabolic disorders, including MetS [10], and play an important role in the development and progression of CVD [4]. The Mediterranean diet (MedDiet) represents a healthy dietary pattern, and has been widely reported to be a model for healthy eating, due to its contribution to a better cardiometabolic profile [11,12]. A meta-analysis of more than 1.5 million healthy adults demonstrated that the adherence to a MedDiet was associated with a significant improvement in health status and a reduction in the risk of cardiovascular-related and overall mortality [11].

The clustering of metabolic risk factors leads to several cardiometabolic disorders, and adherence to a MedDiet in conjunction with muscular fitness (MF) is considered an important marker of metabolic health. In accordance, considerable effort is needed to better understand the underlying link between muscular strength, adherence to a MedDiet, and related co-morbidities. The present study therefore aimed to investigate the combined effect of adherence to a MedDiet and MF on cardiometabolic health in young adults from Colombia.

## 2. Methods

### 2.1. Study Design and Sample Population

The present cross-sectional study was based on a relatively large-scale project (FUPRECOL, in Spanish: Fuerza Prensil Colombia, Association between Muscular Strength and Metabolic Risk Factors in Colombia) consisting of 1248 young adults [13] between the ages of 18 and 30 years old, who were of low to middle socioeconomic status (SES; 1–4 on a scale of 1–6, as defined by the Colombian government) and enrolled in public or private universities in three distinct areas of Colombia: the capital district of Bogota (Cundinamarca), Tunja (Boyacá), and Santiago de Cali (Valle del Cauca). All participants, in addition to their parents/guardians, provided written informed consent. Each study was approved by the authorized Institutional Review Board (UMB N° 01-1802-2013), and complied with the Declaration of Helsinki (as revised in Hong Kong in 1989 and in Edinburgh, Scotland, in 2000). The present study was also in consonance with Colombian laws regulating clinical research on human subjects (Resolution 008430/1993 of the Ministry of Health). Exclusion factors included a clinical diagnosis of CVD, diabetes mellitus 1 and 2, pregnancy, the use of recreational drugs and, in general, the presence of any disease not directly associated with nutrition. Volunteers received no compensation for their participation.

Data regarding the ethnicity, habitual physical activity, smoking, and alcohol consumption were collected using a questionnaire, in the presence of trained personnel to answer any questions that the subjects might have had. Ethnicity was divided into the following subgroups: (a) Indigenous (Indigenous ethnicity included 1,378,884 (3.4%) individuals belonging to various groups, i.e., Emberá, Misak, Ika, Kankuamo, Nasa, Wayuu, Awuá, and Mokane), (b) Black or Afro-Colombian, or (c) others (e.g., Mestizo). Mestizo Colombians have mixed European and Amerindian ancestry, and are the largest ethnic group in the country (49–58% of the population). Habitual physical activity was assessed as an accumulated time of 150 min/week or more, undertaking moderate- to vigorous-intensity physical activity, which was considered to meet physical activity recommendations for adults. Smoking and alcohol consumption was divided into the following subgroups: non-smoker or smoker (defined as those with a daily consumption of 20 or below), and non-drinker or drinker (>5 alcohol units/week).

### 2.2. Adherence to a Mediterranean Diet

To assess the degree of adherence to a MedDiet, a KIDMED index (Mediterranean Diet Quality Index) questionnaire was used [14], which is based on 16 self-administered questions that sustain the principles of MedDiet patterns and those that undermine it. The final results of the index varied between 0 and 12 points; the questions that have one negative connotation in relation to MedDiet were equal to −1, and those that constitute one positive aspect were equal to +1. Participants were classified into the following three levels: (1) ≥8 points, optimal MedDiet; (2) 4–7 points, improvement needed to adjust intake to Mediterranean patterns; (3) ≤3 points, very low MedDiet quality. For further analysis, we combined levels 2 and 3 and classified it as low adherence to a MedDiet (Group 2). Group 1 was considered optimal adherence to a MedDiet.

### 2.3. Muscular Fitness

The muscular protocols were appropriate for use with this age group and have acceptable levels of validity and reliability for handgrip strength [15], standing broad jump, and vertical jump [16]. We used the standing broad jump, vertical jump, and isometric handgrip dynamometry as indicators of lower and upper body muscular fitness, respectively. For handgrip strength, the subjects were instructed to gradually and continuously tighten their grip for at least 2 s, using the ideal palm grip. The test was performed twice, alternately, with both hands. Scores were calculated as the average of the right and left handgrip strength and were recorded in kilograms (kg), without consideration of hand dominance. This test was assessed using a digital dynamometer with an adjustable grip (Model T-18 TKK SMEDLY III^®^, Takei Scientific Instruments Co., Ltd., Niigata, Japan).

The standing long jump was assessed using the maximum horizontal distance reached. The participants flexed their knees, held their arms forward and parallel to the ground, and jumped without momentum. The test was performed twice, and the best attempt was recorded. The distance in centimeters (cm) was measured by the appraiser, whom was positioned next to the participant, from the scribe line to the point where the back of the heel touched the ground. The vertical jump height was assessed with subjects stood with their feet and toes on the measurement mat (Takei 5414 JUMP-DF DIGITAL VERTICAL^®^ Takei Scientific Instruments Co., Ltd., Niigata, Japan). The participants performed a countermovement jump. Two jumps were performed with one minute allowed for recovery between attempts. The height in cm was measured.

### 2.4. Blood Sampling

All blood samples were collected, after fasting for a period of 10–12 h, at a mobile clinic by certified health professionals. Participants were asked not to engage in prolonged exercise during the 24 h prior to testing. The biochemical profile included the following: (i) high-density lipoprotein cholesterol (HDL-C); (ii) triglycerides; (iii) low-density lipoprotein cholesterol (LDL-C); (iv) total cholesterol; and (v) glucose fasting by enzymatic colorimetric methods. 

We calculated a composite MetS score (MetScore) that reflects a continuous score of the five MetS risk factors. The MetScore was calculated from the individual subjects’ data, based on the Harmonizing International Diabetes Federation [17], and standard deviations were calculated using data from the entire subject cohort at baseline. The equation used was MetScore = ([HDL-C: ♂ ≤ 40 or ♀ ≤ 50 mg/dL]/SD (standard deviation): ♀ = 1.04 or ♂ = 0.19*[−1]) + ([triglycerides: 150 mg/dL]/SD: ♀ = 0.99 or ♂ = 0.97) + ([fasting glucose: 100 mg/dL]/SD: ♀ = 0.98 or ♂ = 1.02) + ([WC: ♂ ≥ 94 or ♀ ≥ 80 cm]/SD: ♀ = 0.99 or ♂ = 0.94) + (Mean arterial pressure: 100 mm Hg]/SD: ♀ = 0.88 or ♂ = 1.07), where MAP represents mean arterial pressure. A subject was diagnosed as having a healthier metabolic profile if he or she met three or more of the following criteria: (1) fasting glucose level ≥ 100 mg/dL; (2) triglyceride level ≥ 150 mg/dL; (3) blood pressure (systolic/diastolic) ≥ 130/85 mm Hg; (4) HDL-C level < 40 mg/dL ♂, and <50 mg/dL ♀; and (5) WC ≥ 90 cm ♂, and ≥80 cm ♀, according to the Harmonizing International Diabetes Federation [17].

### 2.5. Physical Examination

Body weight (kg) was measured using an electric scale (Model Tanita^®^ BC-418^®^ SportLife, Tokyo, Japan) and height (cm) with a portable stadiometer (Seca^®^ 216 SportLife, Hamburg, Germany). Body mass index (BMI) was calculated as weight (kg)/height (m^2^). WC (cm) was measured as the narrowest point between the lower costal border and the iliac crest using a metal tape measure (Lufkin W606PM^®^ SportLife, Parsippany, NJ, USA), in accordance with the guidelines of the International Society for the Advancement of Kinanthropometry [18]. The technical error of measurement was less than 2% for all anthropometric variables. We measured blood pressure levels on the left arm at the heart level using an automatic device, the Omron M6 Comfort (Omron^®^ Healthcare Europe B.V., Hoofddorp, The Netherlands). Participants were seated in a semi-reclined position with arms relaxed and supported, and with the midpoint of the arm at the level of the heart. The mean arterial pressure (MAP) was calculated using the following formula: MAP = (systolic blood pressure + (2 × diastolic blood pressure))/3.

### 2.6. Data Management

The results of the standing broad jump, standing vertical jump, and isometric handgrip tests were transformed to standardized values (*z*-scores) for the whole sample. The sum of the *z*-scores of the three tests was calculated to create the MF score. Participants were divided into two groups: low MF group (first tertile) and high MF group (second and third tertiles), as previously described [19]. Several studies have shown that the least-fit tertile has the strongest association with a poor cardiometabolic profile and high mortality rate [7,13], suggesting that this could be considered the unfit group. Subsequently, according of the adherence to a MedDiet (optimal and low) and muscular fitness (high and low), four exclusive groups were created: (1) Low MF/Low Adherence to a MedDiet, (2) High MF/Optimal Adherence to a MedDiet, (3) Low MF/Optimal Adherence to a MedDiet, and (4) High MF/Low Adherence to a MedDiet.

### 2.7. Statistical Analysis

Descriptive data are presented as the mean and standard deviation. An independent two-tailed *t*-test for continuous variables and the Chi-squared test for categorical variables were used to examine gender differences. Analysis of covariance (ANCOVA) stratified for gender and adjusted for age, university, ethnicity, and tobacco use by Bonferroni’s post-hoc multiple comparison test was used to assess the differences between the mean values of cardiometabolic markers across the four combined groups of adherences to a MedDiet and MF.

Binary logistic regression models stratified by gender and adjusted for age, university, ethnicity, and tobacco use were constructed to verify the association between lower cardiometabolic risk and the combined groups of adherence to a MedDiet and MF. Analyses were performed using SPSS (Statistical Package for the Social Sciences for Windows, version 21.0 SPSS Inc., Chicago, IL, USA). A *p* value of <0.05 denotes statistical significance.

## 3. Results

### 3.1. Study Participants

The results obtained for the anthropometric variables, biochemical profile, blood pressure, muscular fitness, and adherence to a MedDiet are shown in Table 1. The final sample comprised a total of 1248 participants. In the cohort of collegiate students, women were found to have a significantly lower WC, triglyceride level, and *z*-score muscular fitness (handgrip (kg), standing long jump (cm), and vertical jump (cm) variables), in addition to a greater HDL-C level and MetS score, than men (*p* < 0.05).

### 3.2. Cardiometabolic Risk Variables across Combined Groups of Adherence to a Mediterranean Diet (Low Adherence vs. Optimal Adherence), and Muscular Fitness (Low MF vs. High MF) by Sex

Female participants classified as having a low adherence to a MedDiet and low MF had, on average, the lowest levels of HDL-C when compared with those with an optimal adherence to a MedDiet and high MF (*p* = 0.001) (Table 2). In addition, participants classified as having an optimal adherence to a MedDiet and low MF had a higher WC when compared with those with a high adherence to a MedDiet and high MF, or a low adherence to a MedDiet and high MF (*p* < 0.001). Moreover, the overall cardiometabolic profile of participants with high MF and a low adherence to a MedDiet, or high MF with an optimal adherence to a MedDiet, was significantly different to that of participants with low MF and low adherence to a MedDiet, or low MF and an optimal adherence to a MedDiet (*p* < 0.001). In men, after adjustments for age, university centre, ethnicity, and tobacco use, male participants classified as having an optimal adherence to a MedDiet and high MF had, on average, the healthiest levels of waist circumference, systolic blood pressure, diastolic blood pressure, HDL-C, and triglycerides, when compared with the group with optimal adherence to a MedDiet and low MF (*p* < 0.02 for all). The optimal adherence to a MedDiet/low MF group showed the highest levels of systole blood pressure and glucose, when compared with those with low adherence to a MedDiet and high MF (*p* < 0.04 for all). In addition, both the high MF with low adherence to a MedDiet and high MF with optimal adherence to a MedDiet groups had significant difference between the low MF with low adherence to a MedDiet group and the low MF with optimal adherence to a MedDiet group (*p* < 0.001 for all).

Participants classified as having an optimal adherence to a MedDiet/high MF, as well as those with low adherence to a MedDiet/high MF had, on average, the lowest MetS score, when compared with those with low MF/low adherence to a MedDiet and those with low MF/optimal adherence to a MedDiet in both sexes (*p* < 0.01 for all). We did not find any difference between high MF/optimal MedDiet and high MF/low MedDiet both in males and females.

### 3.3. Association between Metabolic Profile, Adherence to a Mediterranean Diet, and Muscular Fitness

As shown in Table 3, both female (odds ratio (OR) = 2.01; 95% confidence interval (CI): (1.8–3.7); *p* = 0.02) and male (OR = 3.38; 95% CI: (1.9–5.8); *p* < 0.001) participants from optimal MedDiet/high MF group had the highest odds of expressing a healthy metabolic profile when compared to those of the low MF/low MedDiet group, after adjustment for age university, ethnicity, and tobacco use.

## 4. Discussion

The main findings of this study suggest that the combination of an optimal adherence to a MedDiet, followed by adequate levels of muscular fitness, seems to provide the highest protection against cardiometabolic risk. Subjects classified as having an optimal adherence to a MedDiet and high MF had the highest odds of expressing a healthier metabolic profile, compared with those with a low adherence to a MedDiet and low MF, in both men and woman. We also found significant differences on the cardiometabolic variables per the different levels of muscular fitness and MedDiet groups. Overall, participants with high MF and optimal adherence to a MedDiet had improved levels of some cardiometabolic variables when compared to those with low MF and optimal MedDiet adherence, as well as those with low MF and low MedDiet adherence, but not when compared to those with high MF with low MedDiet adherence, in both sexes. 

Our findings suggest the existence of a tight relationship between an adherence to a MedDiet and MF on the cardiometabolic health of collegiate students. Collectively, these findings suggest that a combined effect of optimal adherence to a MedDiet in the presence of a healthy MF profile seems to improve the health status. However, it seems that the deleterious consequences ascribed to the low adherence to a MedDiet process could be counteracted, to some extent, by maintaining appropriate levels of muscular fitness. 

The mechanisms through which muscular fitness leads to reduced cardiometabolic risk incidence remain incompletely understood. The skeletal muscle constitutes the largest insulin-sensitive tissue in the body, and is the primary site for insulin-stimulated glucose utilization [20]. Given that muscle mass accounts for 85% of the body’s glucose disposal in the postprandial state, it plays a crucial role in maintenance of systemic glucose metabolism [21]. Additionally, high levels of muscular fitness have been associated with decreased body fat and increased insulin sensitivity, with improved ß-cell function and increased basal metabolic rate, among others [22,23]. Currently, studies have postulated that muscular strength might have a stronger influence on the components of metabolic syndrome [24], although both have been recognized as independent protectors against cardiovascular disease [25,26] and overall mortality [7]. 

Accumulated evidence suggests that a Mediterranean diet can play an important protective role against development of MetS through several pathways [10,11]. In addition, guidelines and recommendations from scientific experts and institutions strongly support the concept that the MedDiet may reduce the risk of several non-communicable diseases [27]. The MedDiet is characterized by a high intake of vegetables, legumes, fruit, nuts, unrefined grains, fish, and monounsaturated fatty acids. These nutrients may influence, through biochemical pathways, a reduction of CVD risk [28,29]. Previously, Kim Knoops TB et al., have reported that a Mediterranean diet rich in plant foods, in combination with at least 30 min of physical activity per day, is associated with a significantly lower mortality rate [30]. In our study, both low levels of MF showed a key association with a high cardiometabolic risk profile, not only in the presence of an optimal, but also of a low adherence to the MedDiet. Collectively, our findings provide new information on the role of MedDiet on cardiometabolic health, but also the key role of MF on college students, and builds on the previous literature, by showing that a combined effect of MF and MedDiet seems to be more effective on metabolic health. 

To the best of our knowledge, this is the first study assessing the combined association of MF and adherence to a MedDiet on cardiometabolic health in college students. Analyzing these associations, we observed that subjects with high muscular fitness and optimal MedDiet adherence showed the healthier metabolic profile. These results have public health and clinical implications, since both diet pattern and muscular fitness are primary modifiable lifestyle factors, and they should be simultaneously considered in the interventions development with the aim of increasing cardiometabolic health. In addition, our results highlight the importance of MF tests (such as handgrip) as an excellent tool for population-based surveillance and health monitoring.

Strengths of this study include the novelty of the analyses of combined associations of MF with MedDiet on the cardiometabolic profile in a large sample of college students, for both sexes. In addition, our findings are important from a public health point of view, because they state that to gain efficiency in the primary prevention of cardiovascular risk, physical activity programmes should include resistance exercises.

This study is subject to certain limitations. First, owing to its cross-sectional design, we cannot infer that our observed associations reflect causal relationships. Second, we gathered information regarding adherence to the MedDiet through the Kidmed questionnaire. Although this is the most widely instrument used to score MedDiet adherence, further validations may be interesting. Third, a previous study conducted in this type of population showed a high incidence of sedentary time [31]; thus, the muscular fitness levels may be lower than expected, which could have influenced the relationship with cardiometabolic risk and optimal adherence to MedDiet.

## 5. Conclusions

Despite the cross-sectional design of our study, we showed that the combination of high levels of muscular fitness and an optimal adherence to the MedDiet is associated with a healthier metabolic profile in college students. However, high levels of muscular fitness seem to counterbalance the deleterious effects of low adherence to the MedDiet.

## Figures and Tables

**Table 1:** Characteristics among a sample of college students from Colombia (mean (SD) or frequency (%)).

Characteristics	All (*n* = 1248)	Women (*n* = 714)	Men (*n* = 534)
Age (year)	20.1 (2.7)	20.1 (20.1)	20.2 (2.9)
Ethnicity (mestizo) %	86.2	86.6	85.7
Weight (kg)	62.3 (12.7)	57.4 (10.4)	69.1 (12.6)
Height (cm)	164.3 (9.1)	158.5 (5.6)	172.2 (6.6)
Waist circumference (cm)	73.6 (9.1)	70.3 (7.9)	78.1 (9.7) *
HDL-C (mg/dL)	40.8 (11.4)	42.5 (12.1)	38.5 (10.2) *
Glucose (mg/dL)	87.7 (10.1)	87.3 (10.5)	88.2 (9.4)
Triglycerides (mg/dL)	90.2 (46.6)	87.1 (43.8)	94.5 (49.9) *
Diastolic Blood Pressure (mm Hg)	72.5 (10.2)	71.7 (9.6)	73.6 (10.9)
Systolic Blood Pressure (mm Hg)	114.4 (12.8)	110.5 (11.1)	119.7 (13.3)
MetS score	−3.70 (2.7)	−3.9 (2.6)	−3.4 (2.9) *
Handgrip (kg)	30.3 (9.5)	23.8 (4.5) *	39.2 (7.1) *
Standing long jump (cm)	139.3 (44.3)	112.5 (24.5) *	175.8 (38.9) *
Vertical jump (cm)	32.7 (9.3)	27.1 (5.4) *	40.4 (7.7) *
*z*-score muscular fitness	−0.1 (2.1)	−0.15 (1.9)	−0.02 (2.3) *
Optimal Adherence to a MedDiet (%)	13.1	14.3	11.4
Meeting PA recommendations	31.1	27.2	35.9 *
Smoking	27.9	26.6	29.5
Alcohol	41.9	35.6	47.6

* Significantly different from woman (*p* < 0.05)—independent two-tailed *t*-tests for continuous variable and Chi-square for categorical variables; MedDiet: Mediterranean diet. PA: physical activity; MetScore: composite metabolic syndrome score.

**Table 2:** Cardiometabolic risk variables for women across combined groups of adherence to a MedDiet (low adherence vs. optimal adherence), and muscular fitness (low muscular fitness vs. high muscular fitness).

Characteristics	High MF/Optimal MedDiet (*n* = 407)	Low MF Optimal MedDiet (*n* = 210)	High MF Low MedDiet (*n* = 73)	Low MF Low MedDiet (*n* = 24)	*F*	*p*
**Women**						
Waist circumference (cm)	69.3 (68.5–70.0) ^b^	72.7 (71.8–73.7) ^a,c^	68.8 (66.7–70.0) ^b^	72.7 (69.9–75.5)	13.2	<0.001
Systolic Blood Pressure (mm Hg)	110.4 (109.4–111.4)	110.4 (108.9–111.8)	109.9 (107.6–12.3)	113.1 (109.0–117.1)	0.5	0.620
Diastolic Blood Pressure (mm Hg)	71.3 (70.1–72.2)	72.7 (71.5–74.1)	70.0 (67.9–72.1)	73.8 (70.3–77.3)	2.4	0.064
HDL-C (mg/dL)	43.3 (42.1–44.4) ^d^	41.1 (39.5–42.6)	44.5 (42.0–47.1) ^d^	36.0 (32.0–40.1) ^a^	5.3	0.001
Triglycerides (mg/dL)	88.0 (83.9–92.1)	86.1 (80.5–90.7)	86.0 (63.0–94.7)	78.8 (63.0–94.7)	0.4	0.713
Glucose (mg/dL)	87.3 (86.3–88.3)	87.6 (86.2–89.0)	86.4 (84.1–88.2)	88.9 (85.1–92.8)	0.5	0.673
MetS score	−4.2 (−4.4–−3.8) ^b,d^	−3.4 (−3.8–−1.9)	−4.4 (−5.0–−3.9) ^b,d^	−2.9 (−3.8–−2.0)	6.1	<0.001
**Men**						
Waist circumference (cm)	75.7 (74.7–76.7) ^b^	82.3 (81.05–83.7)	75.9 (73.2–78.7)	82.07 (78.6–85)	23.4	<0.001
Systolic Blood Pressure (mm Hg)	118.8 (117.4–120.2) ^b^	122.2 (120.2–124.1) ^a^	117.6 (113.7–121.4)	118.7 (112.8–122.6)	3.3	0.019
Diastolic Blood Pressure (mm Hg)	72.3 (71.2–73.4) ^b^	76.4 (74.9–77.9) ^a,c^	70.8 (67.7–73.9) ^b^	74.0 (70.1–77.9)	7.0	<0.001
HDL-C (mg/dL)	39.7 (38.6–40.7) ^b,d^	37.0 (35.5–38.5) ^a^	39.7 (36.8–43.0) ^d^	32.4 (28.5–36.1)	6.2	<0.001
Triglycerides (mg/dL)	89.0 (83.8–94.2) ^b^	106.5 (99.3–113.6)	87.6 (72.7–102.5)	91.5 (73.3–109.7)	5.3	0.001
Glucose (mg/dL)	88.0 (86.9–89.0)	89.1 (87.7–90.5) ^c^	84.3 (81.4–87.2)	89.2 (85.6–92.8)	2.9	0.033
MetS score	−4.3 (−4.5–−3.8) ^b,d^	−2.3 (−2.7–−1.9) ^a,c^	−4.6 (−5.4–−3.7) ^b,d^	−2.5 (−3.5–−1.5)	19.3	<0.001

Data are mean and confidence intervals adjusted for age, university, ethnicity and tobacco use; ^a^ Significantly different from high MF/optimal MedDiet; ^b^ Significantly different from low MF/optimal MedDiet; ^c^ Significantly different from high MF/low Diet; ^d^ Significantly different from low MF/low Diet; MF: muscular fitness; MedDiet: Mediterranean diet.

**Table 3:** Odds ratio of having a healthier metabolic profile by adherence to a Mediterranean diet and muscular fitness.

Parameter		OR * (95% CI)	*p*
Women	Low MF	1	
	Low MedDiet		
	High MF	2.01 (1.8–3.7)	0.020
	Optimal MedDiet		
	Low MF	1.12 (0.4–3.1)	0.820
	Optimal MedDiet		
	High MF	2.1 (0.5–7.0)	0.241
	Low MedDiet		
Men	Low MF	1	
	Low MedDiet		
	High MF	3.38 (1.9–5.8)	<0.001
	Optimal MedDiet		
	Low MF	0.33 (0.1–2.0)	0.246
	Optimal MedDiet		
	High MF	2.16 (0.7–6.5)	0.172
	Low MedDiet		

OR: odds ratios; CI: confidence interval; 1: reference category. * Adjusted for age, university, ethnicity, and tobacco use. MF: muscular fitness; MedDiet: Mediterranean diet.

## Abbreviations

The following abbreviations are used in this manuscript:

MetS	Metabolic Syndrome
MF	Muscular Fitness
CVD	Cardiovascular disease
MedDiet	Mediterranean diet
